# The Prioritization of Lean Techniques in Emergency Departments Using VIKOR and SAW Approaches

**DOI:** 10.4314/ejhs.v31i2.11

**Published:** 2021-03

**Authors:** Mohammad Aminjarahi, Mohsen Abdoli, Yasin Fadaee, Fatemeh Kohan, Sajjad Shokouhyar

**Affiliations:** 1 Faculty of Management and Accounting, Shahid Beheshti University, Tehran, The Islamic Republic of Iran; 2 Department of Industrial Engineering, Faculty of Engineering, University of Qom, Qom, Iran

**Keywords:** Lean techniques, Emergency Department, VIKOR, SAW

## Abstract

**Background:**

Considering various researches were carried out to implement Lean techniques in healthcare centers, this study has tried to investigate how lean principles could be prioritized in the Emergency Department (ED) by comparing physicians and nurses viewpoints.

**Methodology:**

In the first stage, relevant Lean techniques and several criteria to evaluate the ED performance were selected by reviewing the literature. Then, weight factors for each criterion were calculated using the Entropy method, and Lean techniques were compared and ranked via a questionnaire by which the physicians' and nurses' opinions were obtained separately. In the last stage, the final ranking of Lean techniques was done using VIKOR and SAW methods as two powerful means of Multi-Criteria Decision-Making (MCDM).

**Results:**

Theory of Constraints (TOC) was selected as the most appropriate principle from the physicians' viewpoints by both decision-making methods. However, according to the nurses' opinions, Jiduka was the best approach by the VIKOR method, while with the SAW method, 5S was chosen as the most practical Lean technique.

**Conclusion:**

This study has illustrated that although all Lean techniques are useable for ED, these techniques' prioritization has a key role in choosing the more suitable Lean approach. Moreover, it provides a chance for the emergency wards to keep down different costs and improve staff and patient satisfaction and the quality of treatment simultaneously.

## Introduction

Nowadays, with increasing the level of public welfare and increasing technological advancements, society and people have higher healthcare quality expectations. Conscious customers instigate change and improvement in all industries in different areas (1). In each hospital, the Emergency Department (ED) is one of the essential wards because it is crucial to save a patient quickly. Longer waiting time in the emergency department may directly affect patients suffering from complications and, on some occasions, may cause death (2). Also, healthcare and treatment costs increase faster than the costs of other products and services, and this drastic rising in different types of cost has made the situation of healthcare centers more critical (3,4). In fact, increased waiting time can be due to a mismatch between supply and demand for emergency services (5). In this regard, plenty of reputable surveys and researches have indicated that the necessity for improving emergency units has been considerably accepted in terms of costs imposed on the patients, fast service, ED overcrowding, and the importance of patient safety (7–10).

There are many popular methods in to improve the performance of ED around the world, such as demand management, critical path, stream mapping, queuing systems, triages emergency severity index, Lean and Six Sigma management methods, bedside registration, statistical forecasting, conceptual and mathematical modelling, discrete event simulation and balanced scorecard (11).

However, Lean manufacturing can be considered as a more attractive approach in ED in order to ameliorate the quality of care (5). Lean thinking refers to a set of principles, techniques and tools that allow any company in any environment to exactly produce the product (physical and the service characteristics of the product) at the amount (in terms of number) and time (not sooner and not later) the customer wants. Of course, it entails offering the product at an unrivaled price while everyone; namely, customers, owners, employees, suppliers and the whole society, benefit (12).

Over the past several years, scientists tried to use different Lean thinking techniques in treatment centers. For example, Hasle *et al.*(14) stated that the lean concept is useful for hospitals, but the lean concept, along with its implementation methods, should be coordinated with organizational complexities and values originating from a treatment center. Vandana Khan(15) reported that Lenox Hill hospital was able to reduce mean time and standard deviation of the CT Scan process. Cichos*et al.* (16) used Lean methodology to assess the impact of optimizing orthopedic instrument trays at a tertiary medical center. Improta *et al.* (17) used Lean thinking in the emergency departments of a hospital to decrease patient waiting time, improving the processes of the flow of patients, and optimizing activities that generate waste. The emergency department, like other departments of the treatment centers, should be considered by Lean thinking. Also, considering that this ward is more crowded than any other hospital wards and the need for high speed and accuracy in this ward, it is very important to choose the most appropriate Lean tool in this ward.

However, no specific priority has been specified for the use of these techniques yet. Lack of resources and other constraints may have led to limitations in using some of these techniques. Therefore, the selection of the best and most effective techniques for the improvement of the quality and productivity of ED seems to be vital. Overall, the main contributions of this study are as below:

1. Presenting an applicable prioritization to employ Lean approaches in ED;
2. Separating and analyzing this prioritization regarding physicians and nurses' viewpoint;
3. Comparing two famous and strong decision-making techniques in this prioritization.

## Methodology

Firstly, ED performance criteria were identified and listed by studying the relevant research to investigate factors affecting the selection of Lean manufacturing techniques in health centers. The result of this pilot study were 14 criteria that played a key role in evaluating ED performance. This number of criteria will make the analysis process difficult and increase assessment errors. Therefore, nine university professors and medical experts were selected among reputable professors in universities who had considerable experiences and publications in Lean approaches and distinguished professional physicians from two public hospitals in Tehran. In order to balance the number of used criteria; experts were asked to prioritize them based on importance and relevance. Finally, out of 14 available criteria, six were selected. In addition, 11 popular Lean techniques were identified in medical centers. Due to the same reasons and using the same process, five relevant techniques were selected using experts' opinion.

**Figure 1:**
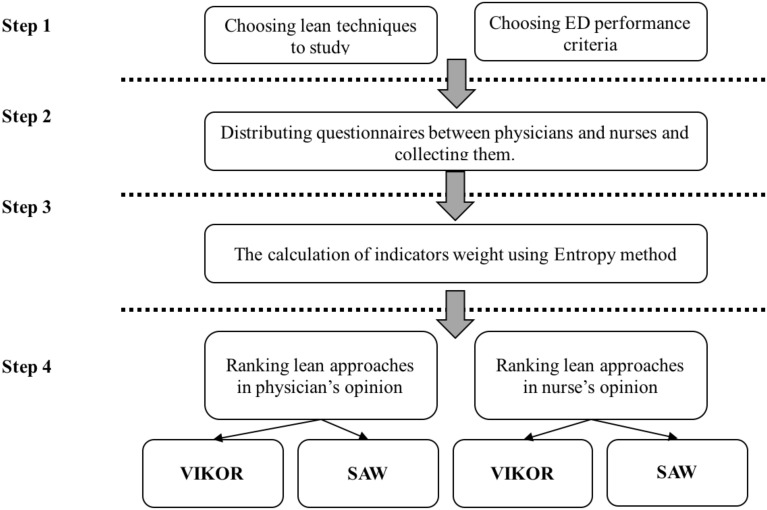
The methodology of this research

The sample consisted of 78 hospital emergency physicians and 87 nurses who had the experience and skills needed to respond to questions and were selected from five large hospitals in Tehran. These hospitals are accounted for as the most crowded and critical ED in Tehran, and there was not a specific plan to implement any Lean principle there (all processes in these centers were doing traditionally) to enhance the quality of services. Using probability sampling methods was not possible due to lack of enough information about the overall population and dispersion of nurses and physician in all ED in Tehran. Furthermore, access to physicians and nurses was limited; hence, the snowball sampling method was used, and new subjects were recruited by existing study subjects. Subjects were asked if they could introduce other physicians or nurses matching wanted conditions and to provide means of connecting with them. Experts' judgment and assessment were referred to in order to ensure the content and face validity of the research tool. The reliability of the questionnaire was assessed using Cronbach's alpha, the value of which was obtained 0.82% using Minitab 16 software. Therefore, the questionnaire is reliable since the value of Cronbach's alpha is higher than 0.70. In order to determine a reliable prioritization for Lean approaches, two strong methods (VIKOR and SAW) have been chosen, which are used in different MCDM problems (18,19). For providing suitable weights for the criteria, Entropy technique is used that is a well-known method in this subject (20). An overview for the procedure of this research is demonstrated in Figure 1. In the following sections, SAW and VIKOR techniques are explained briefly.

**SAW method**: The detailed calculations of SAW are as follows (23):

- Step 1: Forming the decision matrix
  Again, the decision matrix is the matrix X consisting of values x_ij_, where x_ij_ is the score of alternative i in criterion j.
- Step 2: Normalizing the decision matrix
  Since the original values obtained in the decision matrix are not comparable, these data should be transformed using normalization methods. One of the normalization methods that is commonly used in the SAW method is Max technique, which is also known as a linear method. In this method, each value is divided by the maximum value observed in that criterion. During this process, if the criterion is of negative nature, e.g., cost, the scores are converted into positive ones simply by dividing the minimum observed value by them.

$$N_{\mathrm{ij}}=\{\begin{array}{c} \frac{x_{\mathrm{ij}}}{x_{j}^{+}},\mathrm{If}\;\mathrm{criteria}\;j\;\mathrm{is}\;\mathrm{of}\;\mathrm{positive}\;\mathrm{nature}\\ \frac{x_{j}^{-}}{x_{\mathrm{ij}}},\mathrm{If}\;\mathrm{criteria}\;j\;\mathrm{is}\;\mathrm{of}\text{ negative }\mathrm{nature} \end{array}$$

Where N_ij_ is the normalized value of the ith alternative for the jth criterion, and x^+^_j_ and x^-^_j_ are the maximum and minimum observed values of x_ij_ in the criterion j, respectively.

- Step 3: Calculating the weighted decision matrix
  This study uses the entropy method to calculate weights for the criteria. In this step, the decision matrix is multiplied by the weights calculated using the entropy method.
- Step 4: Calculating SAW scores

Final ranking scores are calculated using the following formula:

$$RS_{i}=\sum_{j=1}^{n}W_{j}^{'}N_{ij}$$

where RS_i_ is the ranking score of the alternative i, W'_j_ is the jth criterion's weight, and N_ij_ is the normalized value of alternative i concerning the criterion. In the SAW method, the ranking score of RS represents the alternative's comprehensive performance, and the alternative with the highest value of Si has the highest ranking.

**VIKOR method**: The steps used for the VIKOR method are as follows (24):

- Step 1: Forming the decision matrix
  Like other methods, the decision matrix is also the matrix X consisting of values x_ij_, where x_ij_ is the score of alternative i in criterion j.
- Step 2: Normalizing the decision matrix using the sum method

In the VIKOR method, the technique used for normalizing the decision matrix is based on the sum of alternative scores in each criterion. The following matrix is used for this purpose.

$$N_{ij}=\frac{x_{ij}}{\sum x_{ij}}$$

Where N_ij_ is the normalized value of the ith alternative for the j_th_ criterion.

- Step 3: Finding the best and worst case in alternatives
  The best and worst cases in normalized alternative scores are called N^+^ and N^-^. If the criterion is of positive nature, e.g., profit, then, N^+^=Max (N_ij_) and N^-^=Min (N_ij_), and if the criterion is of negative nature, e.g., cost, then, N^+^= Min (N_ij_) and N^-^= Max (N_ij_).
- Step 4: Calculating the utility and regret There are two basic concepts in the VIKOR method: utility (S) and regret(R). S is the distance of alternative i from the best case, and R is the maximum regret of alternatives from the best case.
  $$\begin{array}{l} U_{i}=\sum_{j=1}^{n}W_{j}^{'}\cdot \frac{N_{j}^{+}-N_{ij}}{N_{j}^{+}-N_{j}^{-}}\\ R_{i}=\max\left[W_{j}^{'}\cdot \frac{N_{j}^{+}-N_{ij}}{N_{j}^{+}-N_{j}^{-}}\right] \end{array}$$
- Step 5: Calculating the VIKOR indexes

The next step is to calculate VIKOR indexes for each criterion using the following formulas. The alternatives are ranked based on the VIKOR index.

$$\begin{array}{l} RV_{i}=v\left[\frac{S_{i}-S^{+}}{S^{-}-S^{+}}\right]+(1-v)\left[\frac{R_{i}-R^{+}}{R^{-}-R^{+}}\right]\\ S^{+}=\max S_{i};S^{-}=\min S_{i}\\ R^{+}=\max R_{i};R^{-}=\min R_{i} \end{array}$$

The constant v is the relative preference toward the utility or regret and is set to 0.5 for this paper.

- Step 6: VIKOR decision conditions

Two final decision conditions in the VIKOR method:

- If alternatives A1 and A2 have the first and second rank in alternatives, respectively, the following formula has to be true:
  $$RV(A2)-RV(A1)\geq \frac{1}{m-1}$$
- Alternative A1 has to be the best choice in either S or R

If none of these two conditions is met, both alternatives are ranked as the same rank.

## Results

After an extensive ED literature survey, eight criteria were obtained to evaluate emergency performance. The criteria include the level of patient's satisfaction, percentage of patients admitted in the ED within six hours, percentage of patients discharged from the ED within 12 hours, percentage of failed CPR, percentage of patients leaving the emergency room, percentage of patients treated in ED, average triage time in each triage level, and the average duration of staying in ED. As mentioned in the previous section, there was a need to lower standards; hence, six criteria that were more important than other indicators were obtained using views of five academic and medical experts.

The six criteria are as follows: Patient's level of satisfaction, percentage of patients admitted in the emergency department within 6 hours, average triage time in each triage level, the volume of patients treated in ED, percentage of failed, and percentage of patients leaving the emergency room.

After selecting the criteria, it is time to select Lean techniques. For this purpose, a total of 11 Lean techniques used at medical centers were obtained using library studies and investigating previous research. These techniques include 5S, Six Sigma, VSM, Kanban, Kaizen, Jiduka, TOC, Poka-yoke, logic tree, seven waste, and matrix *x* which are used in (1, 25–31). Finally, using experts' opinions, five techniques that were more applicable in ED were selected. These techniques are 5s, VSM, Jidoka, TOC, and Kaizen.

In this part of the paper, selected techniques are briefly defined to conceive Lean approaches properly.

- 5S: This word is actually the initials of five Japanese words that represent the five stages comprising this method, including Seiri (classify), Seiton (streamlining), Seiso (cleaning), Seiketsu (standardize), and Shitsuke (sustain) (32).
- Value stream mapping (VSM): It is a tool used to distinguish non-value adding processes from the value-adding processes. This method analyzes the entire processing time and specifies value-lacking times (33).
- Jiduka: It refers to the time when the process is stopped due to a problem. This method requires staff to have maximum authority (34) to solve problems quickly.
- Theory of Constraints (TOC): It is a process that focuses on critical points in a process and tries to solve the constraints that stand in the way of achieving a goal (35).
- Kaizen: It means continuous improvement. This method refers to eliminating wastes on an ongoing basis (26).

In the next step, the target clinical people were asked to complete questionnaires prepared based on comparisons of Lean approaches with respect to each criterion, as well as prioritizing indicators against each other. Finally, a total of 42 and 61 complete responses were obtained from physicians and nurses, respectively, because some of the questionnaires, which were completed imperfectly and inaccurately, were excluded. The summarized demographic information of the final sample is shown in Table 1. After completing questionnaires and exporting data from them, qualitative data were converted into quantitative ones. Then, the geometric means of the data were calculated to be useable in VIKOR and SAW method, and these values were introduced to VIKOR and SAW steps.

**Table 1:** The demographic information of the final sample

Variable	Description	Physicians		Nurses	
		
		Frequency	Percentage	Frequency	Percentage
Gender	Male	16	38	14	23
	Female	26	62	47	77
Age	30 or less	16	38	31	51
	31–39	8	19	24	39
	40–49	15	36	5	8
	50 +	3	7	1	2
Education	Associate's degree or less	0	0	3	3
	Bachelor's degree	0	0	34	55
	Master's degree	0	0	25	40
	Doctorate or higher	42	100	2	2

In the following step, the mean of the weights given by ED physicians and nurses to each criterion is introduced in the entropy method, and the weight for each criterion is calculated and shown in Table 2.

**Table 2:** Final weights using the Entropy method

Criterion	Physicians	Nurses
The volume of patients treated in ED	0.08045	0.1683
Triage average time in triage each level	0.176355	0.031
Percentage of patients admitted in the emergency department within 6 hours	0.102649	0.2817
Patient's level of satisfaction	0.279796	0.1104
Percentage of failed CPR	0.313161	0.3325
Percentage of patients leaving the emergency room	0.047588	0.0761

In the next step, based on the weights calculated by the entropy method and using the normalized decision matrix, the final weight of each alternative was calculated using the SAW method. Considering different situations of work for nurses and physicians, the prioritization for these groups was done separately. The results of these calculations are shown in Table 3.

**Table 3:** Results of the SAW method based on physicians' and nurses' opinions

	C1	C2	C3	C4	C5	C6	SAW	Rank
physicians opinions								

**5s**	0.777778	0.533333	0.555556	0.666667	0.85	0.777778	0.703387	3
**VSM**	1	0.8	0.777778	0.888889	0.7	1	0.816881	2
**Jidoka**	0.722222	0.666667	0.666667	0.555556	0.55	0.666667	0.603512	4
**TOC**	0.944444	1	0.833333	1	1	0.944444	0.975779	1
**Kaizen**	0.666667	0.866667	1	0.444444	0.45	0.611111	0.603482	5

nurses' opinions								

**5s**	0.986733	1	0.572221	0.938889	0.894693	1	0.835501	1
**VSM**	0.614544	0.439677	1	0.564114	0.412807	0.567232	0.641461	4
**Jidoka**	0.705542	0.836167	0.686667	0.646556	1	0.912667	0.811432	2
**TOC**	1	0.487644	0.766355	0.956647	0.661325	0.793778	0.785210	3
**Kaizen**	0.355568	0.441456	0.860496	0.361187	0.533333	0.456693	0.567892	5

**Figure 2:**
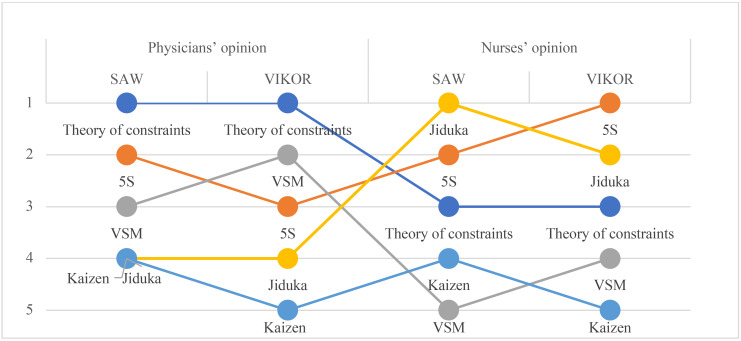
The final ranking of lean techniques for emergency departments

In the following step, after deriving the weights of criteria, the VIKOR method's calculations are implemented. Like former prioritization, physicians and nurses are separated in the decision-making process to find more transparent evaluation for improving ED. The utility (S) and regret (R) values, as well as the VIKOR value for each alternative, are shown in Table 4. Moreover, all priorities of lean approaches based on nurses' and physicians' viewpoints are summarized in Figure 2 to obtain an overall comprehension of the results.

**Table 4:** Results of the VIKOR method based on physicians' and nurses' opinions

	Physicians' opinion			Nurses' opinion		
	
	Utility (S)	Regret (R)	VIKOR value (Q)	Utility (S)	Regret (R)	VIKOR value (Q)
**5s**	0.401268	0.170815	0.470996	0.264855	0.139641	0.175908
**VSM**	0.606318	0.176355	0.635532	0.965472	0.841167	0.976445
**Jiduka**	0.756855	0.256223	0.895476	0.065934	0.028143	0
**TOC**	0.092692	0.04079	0	0.471233	0.335714	0.405523
**Kaizen**	0.723795	0.313161	0.975112	0.635745	0.881362	0.816724
**Max** =	0.756855	0.313161		0.965472	0.881362	
**Min** =	1. 0.092692	0.04079		0.065934	0.028143	

As shown in Table 4, TOC has the least value in all three indexes; thus, it is chosen as the best alternative from the physicians' viewpoint. The 5S and VSM methods are ranked second and third, respectively, in all indexes; so, these methods are chosen as the second and third-ranked alternatives. However, Jiduka and Kaizen have different ranks in two indexes. These two alternatives receive the same ranking based on the first rule of VIKOR. According to the SAW method results in Table 3 for physicians' opinion, TOC is the best choice as the prior method. Still, VSM is known as a better alternative in comparison with the 5S method. Besides, compared with Kaizen, Jiduka can more significantly affect the performance of ED in this decision-making method. However, nurses' opinion is entirely different. Final ranking with the VIKOR method shows that nurses believe that Jiduka can be the best alternative to improve ED productivity and that VSM has the last priority in this classification. Although the SAW approach results demonstrate that 5S is more appropriate than Jiduka in both assortments, these two approaches (5S and Jiduka) have a higher ranking than TOC, which was the first choice of physicians.

## Discussion

The reason for choosing TOC in physicians' viewpoints is that physicians think the problems and constraints imposed on treatment processes are the main reason behind the low productivity. Therefore, addressing and resolving excessive regulations and wasteful constraints can be a good way of moving toward a leaner center. On the other hand, regarding choosing Jiduka from nurses' viewpoints, nurses feel that if they are given more authority and attention, they will play a significant role in improving the ED. Moreover, maybe the difference in weighting ED indicators is the cause of this discrepancy. After C5, which is about failed CPR in ED, physicians have more attention to patients' satisfaction (C4) while nurses focus on treating more patients in ED (C3). Considering the negative effects of pausing a process to solve an error or problem on patients' satisfaction, probably physicians preferred to choose a technique without a direct effect on treatment procedure. Hence, TOC can be accounted for as a more suitable technique in their opinion because it usually considers long-term plans to eliminate constraints in ED. Considering the increasing attention to public healthcare and the need for increasing the quality of treatment services, moving toward creating new and efficient systems in emergency wards is inevitable. New systems in the emergency wards should keep down different costs and improve staffs' and patients' satisfaction and the quality of treatment simultaneously. The lean approaches, while facilitating the achievement of stated objectives, can ameliorate the value of the treatment received by patients. In this regard, there are numerous hospitals throughout the world taking advantage of lean principles in their emergency department. Because of the sensitivity of the ED as well as the high patient traffic in this ward, it is really difficult to make an optimal decision about selecting the right lean approach, due to resource constraints, to improve the performance of this ward. Therefore, the prioritization of these techniques is necessary and unavoidable. It is worth mentioning that, generally, the best classification of Lean principles does not exist. Hence, considering the similarity of structures and procedures in emergency wards, the Lean approaches have been prioritized just for ED in this research.

It should be mentioned that it is impossible to use Lean thinking techniques in the ED without cooperation between managers and emergency personnel. Therefore, promoting Lean thinking culture among staff and the management and their collaboration are essential to execute these approaches.

Even though many studies have been conducted on the implementation of Lean techniques in the emergency department, there are considerable opportunities for the next researches in this area. For example, one of the appropriate areas for future research is to investigate the relationship between criteria and techniques through ANP (analytical network process) or other novel MCDM techniques. It is also recommended to present appropriate models and roadmaps in order to implement different Lean principles in the ED. Also, prioritization of Lean techniques in other wards of the health centers can be another appropriate topic for future research.
